# Illuminating the transitional habitus of the early career health science professional as postgraduate supervisor

**DOI:** 10.4102/hsag.v26i0.1660

**Published:** 2021-10-19

**Authors:** Jeanette E. Maritz

**Affiliations:** 1 Department of Health Studies, Faculty of Human Sciences, University of South Africa, Pretoria, South Africa

**Keywords:** development, habitus, identity, professional, postgraduate, transitional, supervisor, visual elicitation

## Abstract

**Background:**

Early career health science professionals often find themselves in a transitional space when moving from a health science professional role to academia. The role of a postgraduate supervisor is especially troublesome. Transitional spaces often bring uncertainty and perceived or real threats, fear, worry, anxiety and stress. Without support, the result could be detrimental to the mental health of the early career as postgraduate supervisors, thereby impacting their professional identity formation.

**Aim:**

To understand the underlying elements that shaped the early career health science professional as postgraduate supervisors’ habitus and how these features play out in their postgraduate supervision practice.

**Setting:**

The research study was carried out at an Open and Distance e-Learning University (ODeL) and a residential university.

**Methods:**

Visual elicitation methods in the form of seven drawings were used as data. Pierre Bourdieu’s concepts of *habitus* and *hexis* were used as a theoretical lens, and structural analysis with analytical memoing was used to interrogate the drawings.

**Results:**

Early career health science professional as postgraduate supervisors’ bodily habitus presented as fragmented or yet to be formed along with other entanglements, such as emotions, language, power and material arrangements.

**Conclusion:**

These features enable policymakers, employee assistance practitioners, educational developers and experienced academics to consider the changes and structural forces that need to be addressed to support early career health science professional as postgraduate supervisors.

**Contribution:**

A creative means of exploring the inner world of the early career health science professional as postgraduate supervisors is undertaken. In doing so, the research article potentially illuminates what has up to now been ‘unsaid’.

## Introduction

Health science professionals (HSPs) may go through several transitions throughout their career; the most noteworthy and often discussed in the literature is the move from the student status to that of a newly qualified health science practitioner (Haplin, Terry & Curzio 2017). The transition from a professional practitioner to academic is, however, often overlooked. Early career HSPs may experience several transitional challenges, such as a clash of cultures (clinical, professional, vs. academia), which challenges their ability to assimilate. They may be competent clinical teachers; however, the demands of formal academic teaching often require alternative pedagogical approaches. These professionals may need to engage (often for the first time) with research, scholarship and postgraduate supervision (Hunter & Hayter 2019). Academia is a second career for many HSPs; they may have a different career trajectory than other academics and often lack a doctoral degree on entry into their academic jobs.

Evidence suggests that a doctoral degree does not prepare one for an academic career or, more specifically, postgraduate supervision (Pitt & Mewburn, 2016), nor does research skills on their own. Many HSPs are still in the thrush of completing their qualifications, given the changed expectations and requirements for career advancement (Webbstock & Sehoole 2016) in higher education. Once appointed as academics, they are tasked to supervise students and sometimes colleagues with no time to learn the ropes or get a ‘feel for the game’ (Maritz & Prinsloo 2019:568).

Transitional spaces may create uncertainty and perceived or real threats, often bringing along fear, worry, anxiety and stress (WHO 2021). Suppose you are not trained or supported in holding anxiety or how to live with uncertainty during transitions, the result could be detrimental to your mental health and, as I would like to argue in this article, the process of identity formation.

Studies have also found that early career postgraduate supervisors generally do not flock to find support (Carter, Kensington-Miller & Courtney 2017), or there may be less support from mentors and coaches (Maritz & Prinsloo 2015). This may lead to significant anxiety and an uncertain professional identity (Philpott 2015). Identity is what Pierre Bourdieu calls *habitus* (1998:25).

Learning and development are never just about the ‘what and how’ of a subject or the skill to turn this knowledge into practice. At its core, learning to ‘be’ (a postgraduate supervisor) is a profoundly ontological identity-forming process (Beijaard 2019; Halse 2011). The literature on the nature of professional identity formation alludes to the importance of understanding the course in any change or development process or at the very least an understanding of the ‘underlife’ of a social practice (Riseborough 1981:373; Trowler 1998:34). These kinds of ‘understage’ features, referring to salient or hidden aspects of reality, enable policymakers, employee assistance practitioners, educational developers and experienced academics to consider the changes and structural forces that need to be addressed to support those that find themselves in these disorientating spaces of transformation.

I was particularly interested in understanding the underlying elements that shaped the early career HSPs as postgraduate supervisors’ *habitus* and how these features play out in their postgraduate supervision practice? I was equally curious to find a means to illuminate the ‘understage’ or unsaid’ aspects. The questions were as follows: what are the underlying elements that shape the early career HSPs as postgraduate supervisors’ *habitus* and how do these features play out in their postgraduate supervision practice? How can drawings be used to illuminate the ‘understage’ or ‘unsaid’ aspects?

Departing from Bourdieu’s notion that the *habitus* and its related structures operate below the level of consciousness and language (Bourdieu 1984), I pondered on the best way that might illuminate the un-thought of or the unsaid. Previous studies investigating postgraduate supervision pedagogy, practice and other ‘under-belly’ issues often depend on surveys or questionnaires (McCulloch & Loeser 2016) and interviews (Carter 2016; Clegg & Gall 1998: Halse & Malfroy 2009; Naidoo & Mthembu 2015) or at times a combination of these methods (Lee 2018). Ellingson (2015:184) called for different options to present ‘thick descriptions of everyday life’. These include efforts that blend more artistic representations with scholarly writing to draw attention to other ways people, sites and practices may be portrayed through various genres. I ventured to explore how visual elicitation might answer this call and bring the early career health professional as supervisors’ *habitus* to the fore and, by doing so, add to the repertoire of methods.

### The role of *habitus* and *hexis* in becoming (a postgraduate supervisor)

In this exploration, I pay attention to the *habitus* and *hexis* of the early career HSPs as a postgraduate supervisor, as it serves to bring together the many filaments that make up the self (identity) of a postgraduate supervisor in the formation and practice of supervision.

According to Bourdieu, *habitus* shapes both the mind (theory) and the body (practice) (Bourdieu 1990), and has both a cognitive and affective dimension (Benzecry 2018). Bourdieu expresses *habitus as* a collection of actors, for example, individuals, groups or institutions that display both a ‘structured and structuring’ dynamic (Bourdieu 1977:72). Individuals, such as the early career supervisor, are ‘structured’ by both past and present circumstances and experiences; this could be educational experiences or their own supervisory experience. It is ‘structuring’, in that one’s *habitus* helps to shape ones’ present (supervisory) practices and ‘designates a way of being, a habitual state’ (especially of the body) (Bourdieu 1977:214) and as such, possibly the future supervisors’ self-identity. This means that the early career postgraduate supervisor is influenced by supervision practices and influences the supervision field. Because every postgraduate supervisor comes to supervision with different experience and experience postgraduate supervision differently, no one(s) *habitus* will remain the same.

*Habitus* has at its centre a *hexis* (the tendency to hold one’s body in a certain way, a posture, an accent, gestures, speech or interactions) and more abstract mental habits or:

[*D*]urable, transposable dispositions … [these] generate and organise practices and representation that can be objectively adapted to their outcomes without presupposing a conscious aiming at ends or an express mastery of the operations necessary to attain them. (1990:52)

Dispositions are thus embedded unconsciously in the *habitus* of the supervisor. Supervisors may internalise structures, perceptions or conceptions and actions (Bourdieu 1977:86) common to the postgraduate supervisory practice (albeit unconscious), turning these into embodied, permanent dispositions of ‘feeling and thinking’ (Bourdieu 2000:93) or ‘bodily *hexis*’. These dispositions (such as thoughts, perceptions, behaviours, actions and routines) are socially learned. *Hexis* thus becomes the socially conditioned body *habitus*, and therefor*e,* has both an ‘inner’ (disposition) and ‘outer’ (the social) form (Moore 2013:101), which shape the present social actions and practices of a supervisor. Not only is the postgraduate supervisor (some) body in the social world of supervision but the social world of supervision is also inscribed in the body of the supervisor.

Although Bourdieu’s concept of *habitus* has been critiqued for its deterministic nature (Reay 2004), *habitus* still carries the possibilities of new imaginings and possibilities for transformation (Bourdieu 2000). Choices are, however, bound by opportunities and constraints of the external environment or *field,* as Bourdieu frames it.

An iterative relationship exists between the *habitus* of the postgraduate supervisor and the *field*. According to Bourdieu, *field* denotes a social space where interactions, transactions and events occur (Bourdieu 2005). Each *field* has its principles, logic and rules. The concept of *field* is neither innocent nor tranquil but functions much like a ‘game’. Bourdieu suggests that *fields* are occupied by agents (people or institutions) like a football game, and what happens in this *field* is boundaried. There are limits on what can or cannot be performed, who plays and does not play, where the players play, and what rules they play. Unlike the football game, the rules for postgraduate supervision might not be explicit or clear, especially to the early career postgraduate supervisor, and the taken-for-granted practices, in supervision ‘goes without saying because it comes without saying …the tradition is [most often] silent’ (Bourdieu 1977:165). This is also known as the *doxa* of the *field*. Therefore, the structure that produced the early career supervisor’s *habitus* also governs the practice (of supervision). These dispositions are relatively durable over time, which must be kept in mind when and where change is needed or comes to play, for example, where supervision models change to meet higher output demands or in the case of the Covid-19 pandemic, the higher education landscape and all related structures, modes and models of teaching and learning.

When *habitus* meets a new or changed *field*, the resulting disjuncture can create either transformation or *hysteresis* (a temporal lag). This disjuncture could leave the early career HSP as postgraduate supervisors feeling ‘like a fish out of water’ (Dumenden & English 2013:1078) with a set of dispositions and bodily comportments that no longer reflect the field’s requirements. Not all are, however, dark and lost. At times, the changes may entice us to look for possibilities and force lines of creativity (Grant 2018). This sentiment is echoed by Smart (2014) who postulated that although transitions may be disruptive, if well managed, they may enable individuals to flourish.

### Uncovering the ‘underlife’ of the postgraduate supervisor

Over the years, researchers have relied on numerous methods to investigate postgraduate supervisors and their supervision practices. Some attempts have been made to illuminate the ‘underlife’ of postgraduate supervisors. Susan Carter (2016) used storytelling and metaphors as a technique to explore supervisor learning, and found that learning to supervise is often an unsettling and tricky business. She used a ‘medieval’ metaphor illustrating the liminal spaces where supervisors find themselves where their skills and methods are inadequate for their pursuits. It became clear that when challenges are ongoing and troubles are unexpected, it forces supervisors to acquire new qualities and skills.

Grant (2018:367) viewed the ‘becoming supervisor’ from a new materialist perspective as an assemblage, similarly finding the process as both developing and an unsettling pursuit. The assemblages form from several diverse parts ‘in composition with multiple objects, practices, spaces, forces and affect’ (p. 357). This lens allows us to view the emergent supervisor in a more mangled orientation between bodies, things and sensations. These ‘things’, such as technology, lays down new expectations between parts (e.g. the supervisor, student and organisations). New rules and binaries are formed during the process along with the emotions that they produce.

What these studies illuminate is the affective politics of supervision in pressured times. The commonly found examples of negative emotions include feeling overwhelmed (Naidoo & Mthembu 2015), impending dread, frustration (Carter 2016) and anxiety (Grant 2018). These emotions erode the confidence of the postgraduate supervisor, and the practice of supervision becomes bewildering and daunting. Collins, Glover and Myers (2020) confirmed the effects of academic work’s emotional labour, especially the uncertainties, anxieties and identity legitimations within the forced changes, especially digitally evolving workspaces. The challenges are more pronounced for early career supervisors as they struggle to balance other academic responsibilities, such as teaching and research (Naidoo & Mthembu 2015). This threshold moment may place early career HSPs as postgraduate supervisors in a liminal space (Rhem 2013) of ambiguity and disorientation that may prove counterintuitive, and thus troublesome.

## Research methods and design

### My situatedness

I situate myself within a postmodern paradigm. The core of postmodernism doubts whether any method or theory, discourse, genre, tradition or novelty has a universal claim as the (in)conclusive ‘right’ or privileged form of authoritative knowledge (Richardson & St. Pierre 2005:961). Conventional methods of knowing are not automatically rejected as false; however, they are open to enquiry and critique. Professionally, I hold a doctorate in Psychiatric Nursing Science; I am a mid-career researcher and academic.

### Study design

This article formed part of a larger project, namely, ‘Developmental Pathways of Supervisors’ and followed an exploratory sequential mixed-methods approach (QUAL-quan) (Fetters & Freshwater 2015). The first qualitative phase followed a phenomenological lifeworld approach (Brooks 2015; Dahlberg, Dahlberg & Nystrom 2019). Phenomenology describes the meaning and significance of experiences (Creswell & Poth, 2018). A lifeworld constitutes a space within which the taken-for-granted assumptions form the basis of how we make meaning. Lifeworlds are fundamentally intersubjective, meaning that a shared understanding helps us to relate one situation to another. I was specifically interested in how these experiences were produced, performed or maintained in the *field*. The findings of the first phase and a literature review directed the survey to the second phase of the project that aimed at understanding how academic, cultural, social and symbolic factors influence the individual *habitus* of the supervisor within the *doxa* of a particular field.

### Setting

This study took place at one of the residential, public universities and the other at an open distance and e-learning (ODeL) public university in South Africa. The ODeL university follows a multi-model approach (virtual and face-to-face contact) to supervision, whereas the residential university follows a face-to-face approach. The predominant practice included a one-on-one supervision model in both institutions situated within metropolitan areas.

### Study population and sampling

Purposeful sampling (Lawrence 2020) was used, and inclusion and exclusion criteria were set (Robinson 2014). The inclusion criteria required participants to have completed at least a Master’s degree, plus 3 to 5 years of supervision experience. A widely accepted definition of an early career academic as advanced by Garbett and Tynan (2010:175) sets a flexible parameter as ‘the first five years after completing a PhD’. According to Dempsey, transitioning from clinician into academic can be anything from 2 to 4 years before early career academics begin to feel settled and familiar with the role (Dempsey 2007). This parameter may differ in different fields and professions. They should have supervised students to completion in order to illicit a complete experience from the initial supervision contact to the submission of a dissertation or thesis for examination.

Participants needed to be full-time employers of a public university. Supervisors in adjunct or external positions or those in private higher education institutions were not approached for this study. The heads of two health science departments at the two universities were approached through electronic communication, explaining the aim of the research and requesting for the permission to approach early career postgraduate supervisors in their departments. The heads of departments approached potentially eligible participants electronically, whilst two additional participants were approached via personal, professional contacts. A total of 16 positive responses were received when requesting interviews, with nine interview participants initially agreeing to construct a drawing and two participants declining without giving a reason. Of the final seven participants, two were men and five were women, with their age range being 29–56 years and a mean age of 43 years. The higher age could be ascribed to professionals choosing academia as a ‘second’ career.

### Ethical considerations

The ethical considerations suggested by Ingham-Broomfield (2017) were taken into account. Consent to conduct research was granted from the two sites where data were collected (2015_RPCS_087_AR & PM-26-5-16). Informed consent was obtained from all participants through a letter communicating the necessary information about the study. Participants agreed that the information provided might be used for research purposes, including dissemination through peer-reviewed publications and conference proceedings. Participants granted permission for the images of the drawings to be used. Confidentiality was maintained through the anonymity of responses. Participation was voluntary, and participants were informed that they could withdraw at any stage of the project without penalty. There were no foreseeable physical risks involved in this study. Emotions may, however, be unpredictable and lingering, thus causing the participant to be vulnerable. I have an advanced qualification in psychiatric nursing and could, therefore, contain, assess and refer where emotional assistance was needed. The participants had the right to stop or reschedule the interview if they felt the need to do so. None of the participants required such interventions.

### Data collection

The qualitative data in the first phase of the project (2016–2017) included nine in-depth individual interviews that took place in the participants’ office. Each participant was asked to provide a drawing before the individual interview; they were free to decline. I initially used the drawings as an ice-breaker to set the stage for discussion. Participants had the opportunity to discuss their drawing; however, the aim was not to analyse the drawing per se. A total of seven participants agreed to produce a drawing. Participants were given only a broad indication of the overall research topic (Joffe & Elsey 2014). This was performed to minimise the undue influence on the participants or constrain the research topic’s scope.

Participants were requested to provide the first thought, word or image that arose from the word ‘supervision’. They were then asked to draw this thought or image by free associations and free choice of colours. A container with 64 coloured pencils was provided as well as blank, unlined paper. The same size of paper was offered to each participant, although one of the participants used lined paper. They were reassured that the drawing was not to test for drawing ability but to connect to the investigation topic. The drawing session lasted from three to 15 min. Participants displayed differences in their willingness and ability to produce drawings. Most participants had difficulty choosing what information or perception to attend to and generally took a while before drawing. Most participants apologised for their lack of drawing ability, and one proclaimed a ‘drawing block’.

### Data analysis

The drawings on their own were not the analytical focus at the time of the interviews. After reflecting on the drawings for some time, their value as a stand-alone data set became apparent. Irwin and Winterton (2011) recognised the validity of multiple produced interpretations of data, and that those different kinds of data could support different interpretations and accounts of data. The authors further concluded that qualitative data often produce a wealth of information not used in the initial reporting of project findings and the value of revisiting previous data that can reveal new meanings or methodological insights.

Bourdieu challenges us to use *habitus* to interrogate the data rather than explaining the data. Miles, Huberman and Saldaña (2014) advocated that interpreting what we see as still visual documentation is a more holistic venture than a systematic one. The authors suggest that analytical memoing of our interpretations about the image is a more suitable form of investigation than a detailed breakdown of components, such as colour, contrast and composition. Although I support this stance, I believe that a systematic approach to include the above elements could add value. Leibowitz (1999) provided some guidance on this matter. The author suggests that one engages with the drawing firstly on an impressionistic level before engaging in a structural analysis. One can engage kinaesthetically, viscerally or affective. These impressions are then recorded. I started by establishing a coding parameter or formal categories, such as picture size, position, shown body details, face size, facial displays, physique, clothes, words, symbols and colours. These formed the basis of a matrix (Miles et al. 2014). After obtaining the objective categories, themes were identified with some subjective memos (Richards 2015). I asked questions such as ‘what is portrayed, what is the position on the paper, is there something to say about the gesture or mimic of the person in the picture?’ (Bock, Iisermann & Knieper 2011). The drawings were divided into four categories:

Category 0: when the drawing was detached from the task (1)

Category P: when the drawing showed a pictogram (4)

Category PM: when the drawing showed a process or map (1)

Category W: when the drawing consisted of words only (1).

The coding and interpretation were complex, challenging and potentially subjective. Two students with Master’s in research psychology (who signed a confidentiality agreement) acted as co-coders. We reached consensus regarding the parameters, themes and potential interpretations of the drawings. I realised that the analysis required skills in visual deconstruction, comparison, analogical transfer, reiterative cycles of reconstruction, revision and recontextualisation. The processes required honed analytical skills and time. As the drawings were analysed as a set, the original participants were not requested to analyse (their own) drawings. They were, however, asked about the drawings during the interviews as a descriptive exercise. Guba and Lincoln (1989) noted that using procedures to establish accurate correspondence carried too positivist implication. An underlying assumption is that an unchanging phenomenon exists, and can be logically and methodically checked and verified. This brings to the fore the pragmatic limitation of member checking. Reilly (2013) discussed several challenges, but of particular note: participants struggle with the abstract synthesis required to compare their contributions to the coded categorical analysis.

### Trustworthiness

The qualitative field has undergone profound philosophical evolution, which increasingly questions the assumptions of trustworthiness (Denzin & Lincoln 2005). I used Stacy’s ‘eight big tent criteria’ as a framework for quality (Stacy & Hinrichs 2017). The framework focuses on ensuring that the study includes a *worthy topic* that is relevant, significant and interesting, which I argued in the front matter. *Rigour* is established through an in-depth discussion of the methodology, meaningful coherence and *sincerity,* including self-reflexivity and transparency. Whilst the participant-produced drawings provided rich data, the interpretation remains lodged in the viewers’ perceptions and world views. Researcher bias and my sense of what is important might have influenced the descriptions. The findings have, however, been recontextualised in the literature and underscore the discoveries. By ‘showing’ the findings rather than ‘telling’ the reader, *credibility* is upheld in addition to the external co-coding process. The results reveal insights that could be useful in other contexts, which demonstrates *resonance*. The conclusion discusses the *significant contribution*.

## Results

Table 1 provides an overview of the coding parameters and the structural analysis. Four participants produced pictograms, one a process map and one participant wrote words or concepts. One of the participants did not render a drawing and explained her position as a ‘mind freeze’. The participant chose a pink pencil by leaving it on the blank page. I provided a short narrative description of the content in line with the parameters in Table 1 before proceeding with a description of the findings and literature control.

**TABLE 1:** Coding parameter matrix.

Coding parameter	Image 1	Image 2	Image 3	Image 4	Image 5	Image 6	Image 7
**Image size (%)**	75	80	80	75	90	90	10
**Position on page**	Centre	Centre	Centre	Center	Top	Center	Center
**Content**	Drawing	Process map	Words	Drawing	Drawing	Drawing	Pencil
**People in the picture**	Yes	No	No	No	Yes (2)	Partial	No
**Shown body details**							
M F U	M	-	-	-	F	-	-
Size of face	Large	-	-	-	Large	-	-
Face attributes	Large eyes	-	-	-	Large eyes	Large	-
	Sharp or pointed nose	-	-	-	No Nose	eyes	-
	No ears	-	-	-	No ears	-	-
	Raggedy mouth	-	-	-	Mouth Hair	-	-
Facial display	Fearful?	-	-	-	Smiling	Frown Tears	-
Direction of view	Full front	-	-	-	Side	Front	-
Head posture	Forward	-	-	-	Forward		-
Physique	Muscular	-	-	-			-
Clothes	Nil	-	-	-			-
Body poise	Hands in the air	-	-	-	Nil	-	-
Gestures	Unprotected	-	-	-	Hand giving a ruler	-	-
Emotives	Fear or uncertain	-	Lonely	-	Seemingly positive	Worried Fear	-
**Text**	Uncertain	Relationship	Share knowledge	-	-	-	-
	-	Empowerment	Role: Coach or mentor to develop student	-	-	-	-
	-	Research		-	-	-	-
	-	Navigation	Gain knowledge	-	-	-	-
	-	Loneliness	Life-long learning (mutual development)	-	-	-	-
	-	Make or break		-	-	-	-
	-	Achievement	Complete thesis successfully	-	-	-	-
	-	Rewards		-	-	-	-
	-	-	Positive outcome	-	-	-	-
**Objects**	-	-	-	-	-	-	-
Tools	-	-	Book	Book Crown Grass	Voicebox	Book Moon Computer	Pencil
Diagrams	-	Yes	-		-	-	-
Symbols	?? next to the head		-		#xy	@	-
**Colours used**	Blue	Blue	-	Blue	-	Blue	-
	-	Red	-	Red	-	-	-
	-	Green	-	-	Green	-	-
	-	Yellow	-	Yellow	Yellow	Yellow	Pink
	-	-	-	-	Brown	Brown	-
	-	-	-	-	Purple	-	-
	-	-	-	-	Pink	-	-
	-	-	Black	-	-	Black	
	-	-	-	-	-	Orange	-

**FIGURE 1:**
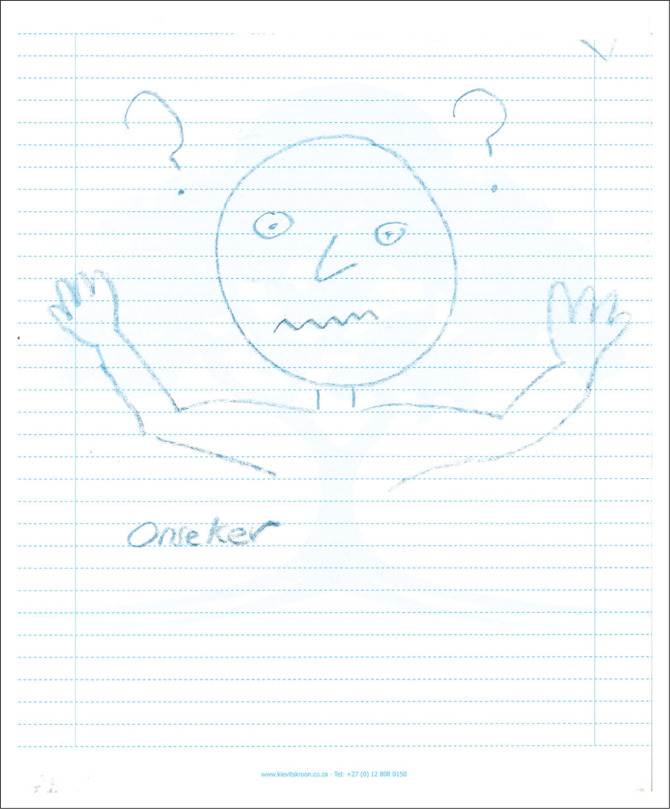
Category P, a pictorial representation of a partial, upper, muscular body in black produced by a participant.

In Figure 1, the head seems rather large when compared with the rest of the body. The eyes are wide, the nose pointed and the mouth ragged. The hands that are upwardly tilted have only four fingers. A single Afrikaans (one of the 11 official languages in South Africa) word is written – ‘*onseker*’, which means ‘uncertain’. Two question marks appear next to the head. Coding memo: the visceral reaction to this picture is one of concerns, apprehensions and worries. Is there potentially a link between the lack of ears and the closed mouth?

**FIGURE 2:**
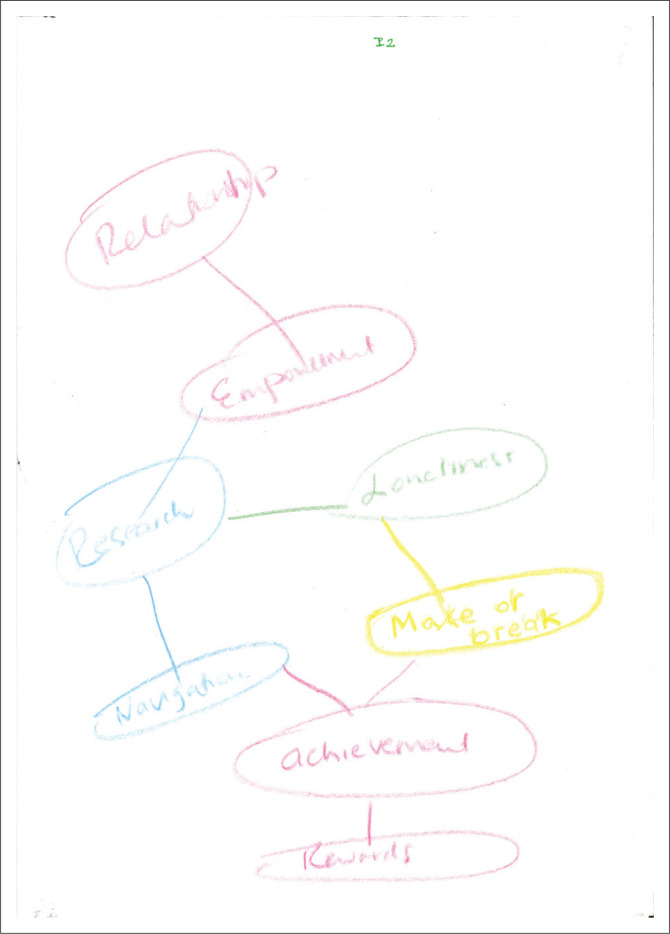
Category PM, a process map of encircled words in black produced by the participant.

In Figure 2, the participant draws a process map where the words are encircled and connected one aspect to the other in a linear fashion. The words include ‘relationship’, ‘empowerment’, ‘research’, ‘loneliness’, ‘navigation’, ‘make-or-break’, ‘achievement’ and ‘rewards’. Coding memo: feeling detached with less of an emotive response as there is no picture. I note the use of the colour black.

**FIGURE 3:**
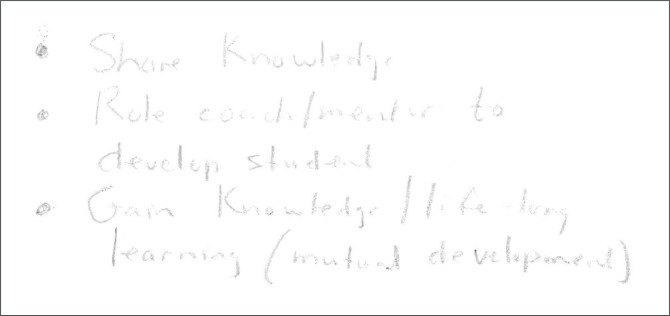
Category W, only concepts are noted in black produced by the participant.

In Figure 3, concepts included ‘share knowledge’, ‘role coach, mentor to develop student’, ‘gain knowledge, lifelong learning, and mutual development’. Coding memo: it is interesting to note that the participants share words rather than drawing; I wonder about this dynamic and the continued use of the colour black.

**FIGURE 4:**
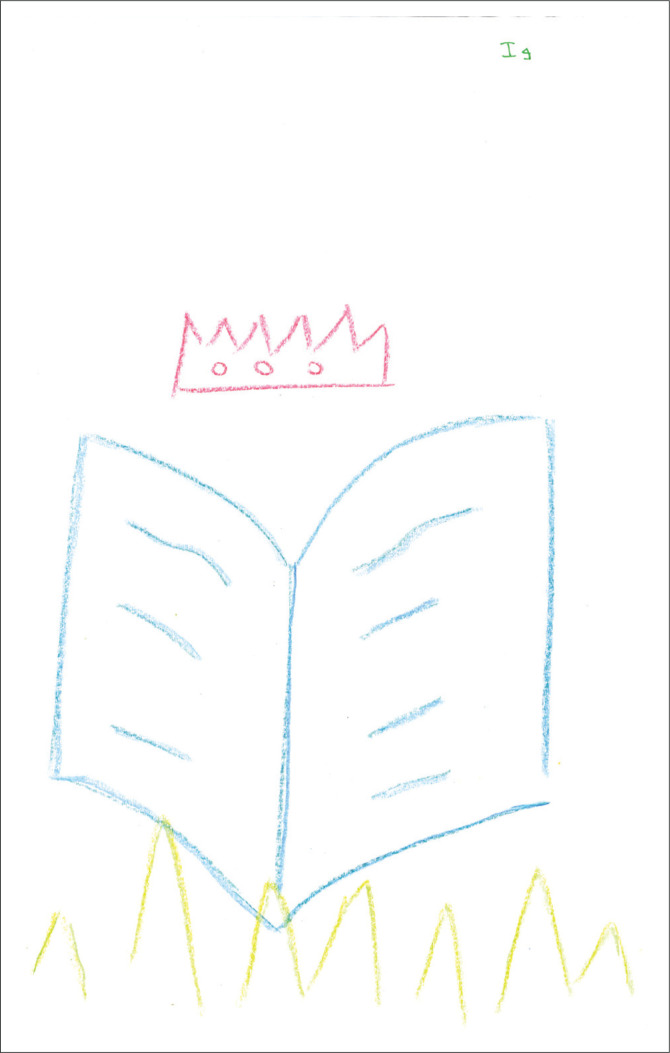
Category P, three objects of different colours produced by a participant.

The pictorial representation in Figure 4 depicts objects such as a crown, a book and grass. Memo note: at last, a colourful depiction. Could this be a positive experience?

**FIGURE 5:**
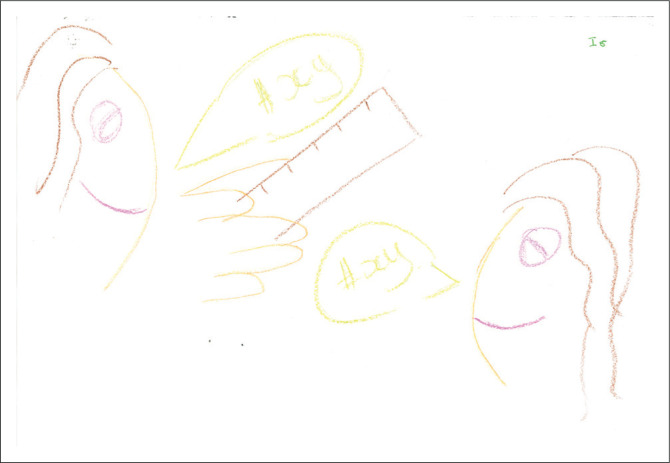
Category P, two heads with hair, eyes and mouth, a ruler and a voice box in colours produced by a participant.

In Figure 5, two female faces are smiling in this pictorial presentation. The two voice boxes may indicate a conversation. An object in the form of a ruler is present. Memo note: the visceral emotion elicited by this picture is more positive. I wonder what is being measured?

**FIGURE 6:**
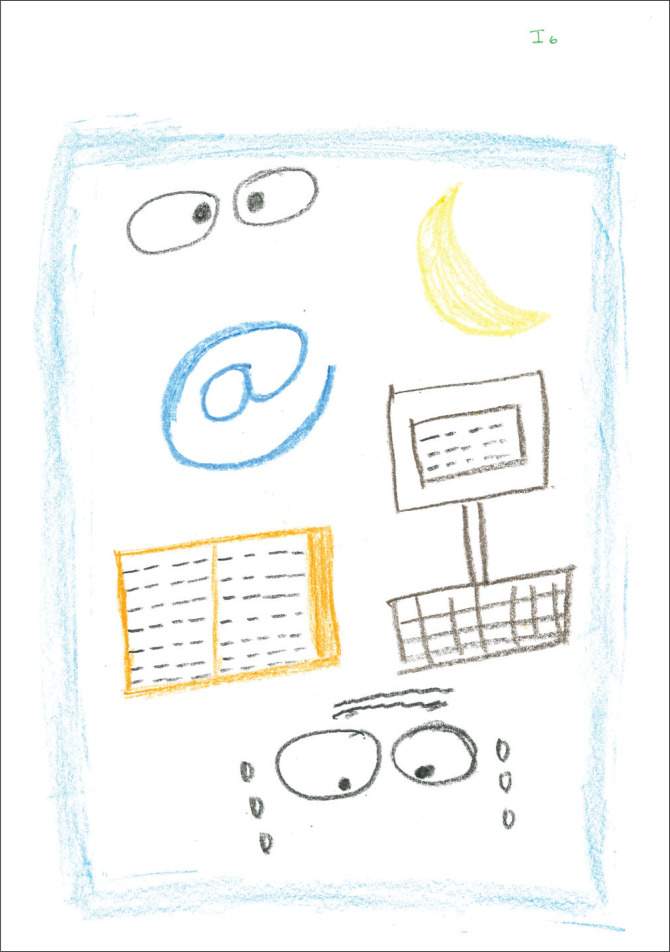
Category PM, multiple pictures and colours produced by a participant.

In this representation, Figure 6, a partial body part, namely, two sets of wide eyes, is presented. One set of eyes are crying, and the forehead has a frown. This picture also has a symbol in the form of an ‘@’ sign. Additional objects include a moon a book, and a computer. Memo note: mixed feelings, the picture is the only pictorial presentation that has a frame, does this indicate some form of containment or possible ‘Pandora’s box’?

**FIGURE 7:**
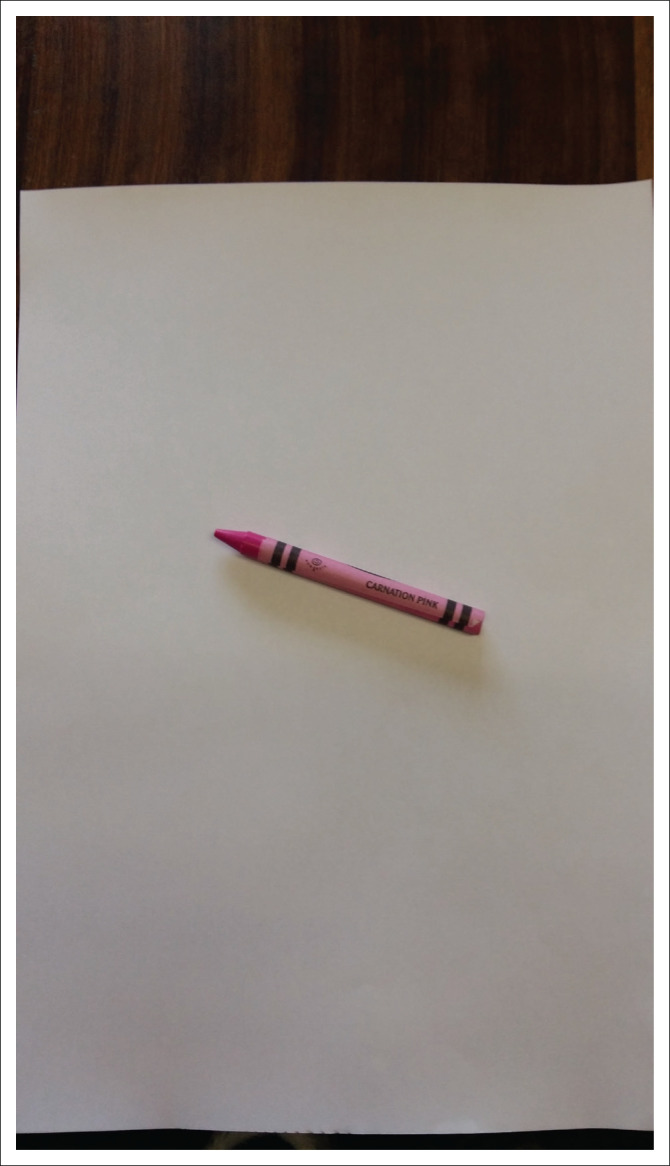
Category 0, the participant could not engage with the task. Drawing reproduced by the author.

In Figure 7, after choosing a colour (pink), the pencil lay untouched on the paper. I reconstructed the image later. Field note: the participant seemed to be nervous and stammered as she reported having a ‘brain freeze’.

## Discussion

In the transformative stage of their professional identity development, early career HSPs as supervisors illustrated ‘what was’ but had yet to form a *habitus* into ‘what was next’. The structural analysis culminated in four main themes, suggesting that the early career HSPs as postgraduate supervisors’ bodily *habitus* is fragmented or possibly yet to be formed. This formation comes along with other entanglements, such as emotions, language, power and material arrangements.

### The body *habitus* is fragmented

Postgraduate supervision practices seem to be bound together by the body, where the body serves as a nexus or interfaces with the practice of postgraduate supervision. The drawing of bodies (Figure 1 and Figure 5) or body parts (Figure 6), such as eyes, seems to have been foregrounded in the pictorial presentations, illustrating how ‘we learn bodily and express our knowledge bodily – all under the organising power of the *habitus*’ (Bourdieu 1977:78). However, this is mainly unconscious and could explain why there has been no mention of ‘the body’ during the interviews (Maritz & Prinsloo 2019). A startling finding is the fragmentation of the presentation of the body of the postgraduate supervisor. There has been no complete pictorial representation of the body; it is instead presented as dismembered parts or has yet to be formed.

### Thinking bodies

The overly large head presented in Figure 1 and the partial body in the form of a head in Figure 5, the depiction of a book in Figure 3, as well as the words, ‘gain knowledge’, ‘share knowledge’ and ‘life-long learning’, may show a greater focus on the cognitive or mental elements of learning and becoming during a transitional period. This might be because of a general cognitive bias in education at large (Tachuchi 2011). Thus, the postgraduate supervisor becomes a thinking (some) body, and the supervision practice becomes embodied. According to Barnett (2009:435), there is an extraordinary and intimate relationship between knowing and being. Therefore, the process of coming to know has ‘person-forming properties’ with life-changing possibilities (Grant 2003). The early career HSPs as supervisors’ body is *in situ*, with momentum, a trajectory, a process and interest with capabilities to transform and become.

### The emotive body

The emotive body proved to be the most powerful theme. Bourdieu (2000) argued that any confrontation between *habitus* and *field* is always marked by affectivity and emotional transactions. The participants attempted to articulate their emotional experiences in words and pictures. Figure 1 has a single comment, stating the experience as ‘uncertain’ with several symbols in the form of question marks. In Figure 6, two large black eyes are depicted with a frown and tears, possibly indicating feelings of fear and anxiety. The moon in Figure 2 may also represent the workings of supervision at night, which might be figuratively or concretely interpreted. The word ‘loneliness’ in Figure 2 provides an additional window to the affective experiences during the early career *habitus* formation.

Emotions expressed were more negative than positive, and were marked by ambivalence and tension. Some describe the university as an ‘anxiety-producing machine’, in which self-expression, emotional connection, immediacy, and enjoyment are now laced with anxiety’ (Hall 2016) and precariousness (Ivancheva 2015). Postgraduate supervising is often portrayed in the literature as a ‘walking on a rickety bridge’ (Grant 1999), simultaneously predictable and unpredictable in its course. The fruit of this emotional labour often manifests itself in feelings of fear and loneliness (Ivancheva 2015) for the early career professional. However, the experience of self-questioning nature of the postgraduate supervisors might have the possibility of moving the *habitus* to a more conscious level and could, therefore, develop new facets of the self.

The smiles on the two faces in Figure 5 might indicate that the relations and processes of supervision, although possibly tricky, have the potential to be pleasurable as well. The crown in Figure 4 and the word ‘reward’ in Figure 2 may equally indicate a process and/or outcome of achievement, satisfaction and gratification. Grant (2003) attested to the dual but not mutually exclusive dynamic in supervision practice of both attendant pleasure and that of risk.

The choice of colours by participants whilst drawing provided additional insights into their potential emotional state. The most predominant colours were blue and yellow (four participants each), followed by two participants choosing red, green, brown and black, and one participant each choosing purple and pink. According to Dael, Perseguers and Marchand (2016), colours ‘carry’ certain affective connotations. However, Gong, Wang and Shao (2017) warned that colour emotions do not exist in an isolated manner. Both blue and yellow could potentially indicate panic or fear (Dael et al. 2016) or show sadness (feeling blue) (Sutton & Altarriba 2016). The subjective experience of anger, danger, failure or threat is often associated with the colour red (Dael et al. 2016; Sutton & Altarriba 2016). Yellow could be related to happiness and positivity (Dael et al. 2016) or fear and cowardness (Sutton & Altarriba 2016). Darker colours, such as black and primarily brown, elicited associations with negative affective states. Black is associated with night, with nothingness and fear of the unknown (Leibowitz 1999). Brown may indicate a sense of security (Leibowitz 1999), whilst the colour green could indicate envy (Sutton & Altarriba 2016) or linked with grass, a sense of aliveness or growth (Leibowitz 1999).

The positioning of the drawings also conveys meaning. Most participants’ pictures covered a significant part of the page. Drawings were mainly found in the centre of the page, with only Figure 5 being placed more to the page’s right. According to Leibowitz (1999), the exact centre placement evokes a feeling of rigidity or needing structure to feel safe. A slight top-right placement in Figure 5 could indicate a movement towards a future position.

### The postgraduate supervisory *habitus*’ *in* relation to power

The placement of the two heads, one above the other, in Figure 5, as well as the words ‘coach and mentor’ in Figure 3, could implicate a particular hierarchical order perceived to be present in the postgraduate supervision relationship or a certain unconscious perception or internalisation of the *doxa* of supervision as adherence to ‘the relations of order’ (Bourdieu 1984:471). According to Grant (1999), institutional pedagogical practices and relations between the supervisor and the student always involve inescapable power relations. The word in Figure 3, ‘sharing knowledge’, indicates that for this participant, supervision should be a collective responsibility of both the supervisor and the student. One should guard against the notion that the student is an empty vessel that must be filled with content (Ivinson 2011), and that learning is, therefore, transactional. The postgraduate supervisor certainly crosses the terrain of a research project *with* a student, and therefore, the relationship has the potential for reciprocity.

### *Habitus* and Language

Concerning the speech bubbles between two (female) bodies with the symbols’ #xy’ inside the bubbles in Figure 5, one might wonder about the nature of what passes between bodies and other processes, relations and arrangements (Mulcohy 2015). Farokhi and Hashemi (2011) noted that:

[*P*]ictures that are symbols recount something profound and complex, so with a consciousness that, itself has limits, it cannot immediately understand everything. It is normal for the symbol (drawing) to carry aspects that are unknown or indecipherable. (p. 2223)

Educational instruction works through various modes, including language, speech and action, acting together as semiotic assemblages. Green (2015) claimed that practices precede practitioners. For the early career postgraduate supervisor or practitioner, the often ‘taken for granted’ professional practices may inform what they say and do, and how practitioners relate to students or others. Without awareness, this ‘taken for granted’ mode may become an entrenched reproduction of discourses and practices, ultimately forming or transforming into a bodily *hexis*.

### Bodies and other material arrangements

Bodies are not held in a vacuum but are understood alongside other material arrangements (such as thesis production, journal articles and grants) and practices (Green & Hopwoo 2015). Early career postgraduate supervisors seemed to have internalised the institution’s corporeal and *doxic* codes (Ivinson 2011; Wacquant 2016), as evident from using words such as ‘achievement, reward, and complete thesis’. The use of symbols such as a ruler in Figure 5 and the process map in Figure 2 might equally refer to the measurement *doxa* that is currently rife in higher education institutions along with other assemblages in which they might be caught up. This illuminates how the current *doxic* structures influence the postgraduate supervisor’s *habitus* and becomes a configuring dynamic in the supervision relationship to the possible exclusion of other measures, such as emotional labour. The danger is that this might predispose the early career postgraduate supervisor to develop a policy-induced *habitus* that might become a durable arrangement and *hexis*. There is evidence of this dynamic when early career supervisors ask for ‘standardised training sessions and guidelines’ (Naidoo & Mthembu 2015) without assuming a critical approach to where and how these training and guidelines inform the current *doxa* as opposed to sound pedagogical approaches that include other activities.

### Supporting the early career HSPs as postgraduate supervisor

Given the fragmented or ‘yet to be formed’ nature of the HSPs as postgraduate supervisors’ *habitus*, it would be critical to remember that *habitus,* by its very nature itself, is in a state of constant flux. Bourdieu writes, ‘*Habitus*, as a product of social conditioning, and thus of a history (unlike character), is endlessly transformed’ (Bourdieu 1994d:7). Its social structures, mechanisms and *habitus* with its socialised bodily states will constantly require adaptation. A change in the *field* necessitates a shift in the *habitus* of the postgraduate supervisor. Educational developers and experienced academics should assist early career HSPs as postgraduate supervisors to understand the significance of external changes and find alternative ways to address change that also impacts the research supervision process (McCallin & Nayar 2012). It also means being familiar implicitly or tacitly, knowing how to do things, such as acting or engaging in different roles and practices or situations or related to various customs, such as sanctions, censures and rewards (Bourdieu & Chartier 2015). Professional developers and experienced academics can supportively induct novices into ‘the rules of the game’ applicable to their particular field.

I would therefore advocate moving beyond training, coaching and mentoring when supporting early career HSPs as supervisors. Both the early career postgraduate supervisor and the professional development teams must develop a psychosocial understanding of *habitus* that allows for a better and richer understanding of how the exterior or broader social structures are experienced and mediated by the interior, the psyche (Reay 2015). In order to prevent the ‘hysteresis effect’, we need to be cognisant that social change occurs because people and institutions pursue strategies that are maladapted to the current state of the *field* in which they act, producing ‘the same of the same’. In supporting early career postgraduate supervisors, it would be necessary for all parties, such as seasoned postgraduate supervisors, professional development specialists and academics, to realise that a (stable supervision) *habitus* is not and should not be a destiny. It is a system of open mechanisms that can constantly be subjected to experience and change, and must be supported throughout their journey, avoiding a one-size-fits-all approach.

## Conclusion

This research article set out to explore the ‘underlife’ experiences of early career HSPs as supervisors through visual elicitation in the form of drawings during a transitional stage of their careers. In the transformative stage of their professional identity development, early career HSPs as supervisors illustrated ‘what was’ but had yet to form a *habitus* into ‘what was next’. Other predicaments, such as uncertainty, ambivalence and tension, became prominent in using language, symbols and additional material arrangements with some potential to be pleasurable as well. This disjuncture can create either transformation or support *hysteresis* (a temporal lag) if they are not supported in dealing with the transitions or managing uncertainty, potentially negatively impacting their identity formation and mental health. I urge policymakers, professional developers and academics to take heed and support early career HSPs as postgraduate supervisors during these transitional spaces in a holistic fashion, including all facets of their *habitus,* especially psycho-social aspects.

Professionals and others finding themselves in transition spaces could use the illuminations to assist their transitioning experiences and potentially decrease their anxiety and stressors.

## Note

In following Grenfell (2012), I adopted the convention of putting Bourdieu’s concepts in *italics* as a reminder, each of which comes with a complex theory of practice and should not be taken as simple analytical metaphors.

## References

[CIT0001] Barnett, R., 2009, ‘Knowing and becoming in higher education’, *Studies in Higher Education* 34(4), 428–440. 10.1080/03075070902771978

[CIT0002] Beijaard, D., 2019, ‘Teacher learning as identity learning: Models, practices, and topics’, *Teachers and Teaching* 25(1), 1–6. 10.1080/13540602.2019.1542871

[CIT0003] Benzecry, C.E., 2018, ‘Habitus and beyond: Standing on the shoulders of a giant looking at the seams’, in T. Medvetz & T.T. Sallaz (eds.), *The Oxford handbook of Pierre Bourdieu Online*, p. 27. Oxford University Press: Oxford, England. 10.1093/oxfordhb/9780199357192.013.25

[CIT0004] Bock, A., Iisermann, H. & Knieper, T., 2011, ‘Quantitative content analysis of the visual’, in E. Margolis & L. Pauwels (eds.), *The Sage handbook of visual research methods*, pp. 265–282, Sage, Los Angeles, CA.

[CIT0005] Bourdieu, P., 1977, *Outline of a theory of practice*, Cambridge University, Cambridge.

[CIT0006] Bourdieu, P., 1984, *Distinction: A social critique of the judgement of taste*, Routledge, London.

[CIT0007] Bourdieu, P., 1990, ‘Structures, habitus, practices’, in P Bourdieu (ed.), *The logic of practice*, pp. 52–65, Polity, Cambridge.

[CIT0008] Bourdieu, P., 1998, *Acts of resistance: Against the new myths of our time*, transl. R. Nice, Polity Press, Cambridge.

[CIT0009] Bourdieu, P., 2000, ‘Making the economic habitus: Algerian workers revisited’, *Ethnography* 1(1), 17–41. 10.1177/14661380022230624

[CIT0010] Bourdieu, P., 2005, ‘*The social structures of the economy*, Polity, Cambridge.

[CIT0011] Bourdieu, P. & Chartier, R., 2015, *The sociologist and the historian*, Polity, Cambridge.

[CIT0012] Brooks, J., 2015, ‘Learning from the lifeworld: Introducing alternative approaches to phenomenon in psychology’, *The Psychologist* 28(8), 264–643.

[CIT0013] Carter, S., 2016, ‘Supervision learning as conceptual threshold crossing: When supervision gets “medieval”’, *Higher Education Research & Development* 35(6), 1139–1152. 10.1080/07294360.2016.1160875

[CIT0014] Carter, S., Kensington-Miller, B. & Courtney, M., 2017, ‘Doctoral supervision practice: What’s the problem and how can we help academics?’, *Journal of Perspectives in Applied Academic Practice* 5(1), 13–22. 10.14297/jpaap.v5i1.235

[CIT0015] Clegg, S. & Gall, I., 1998, ‘The discourse of research degrees supervision: A case study of supervisor training’, *Higher Education Research & Development* 3(17), 323–332. 10.1080/0729436980170305

[CIT0016] Collins, H., Glover, H. & Myers, F., 2020, ‘Behind the digital curtain: a study of academic identities, liminalities and labour market adaptations for the ‘Uber-isation’ of HE’, *Teaching in Higher Education*. 10.1080/13562517.2019.1706163

[CIT0017] Dael, L., Perseguers, M. & Marchand, C., 2016, ‘Put on that colour, it fits your emotion: Colour appropriateness as a function of expressed emotion’, *The Quarterly Journal of Experimental Psychology* 69(8), 1619–1630. 10.1080/17470218.2015.1090462 26339950

[CIT0018] Dahlberg, H., Dahlberg, K. & Nystrom, M., 2019, ‘Open and reflective lifeworld research: A third way’, *Qualitative Inquiry* 26(5), 458–464. 10.1177/1077800419836696

[CIT0019] Dempsey, L.M., 2007, ‘The experiences of Irish nurse lecturers role transition from clinician to educator’, *International Journal of Nursing Education Scholarship* 4(1), 1–14. 10.2202/1548-923X.1381 17542779

[CIT0020] Denzin, N. & Lincoln, Y., 2005, ‘Introduction: The discipline and practice of qualitative research’, in N. Denzin & Y. Lincoln (eds.), *The Sage handbook of qualitative research*, 3rd edn., pp. 1–32, Sage, Thousand Oaks, CA.

[CIT0021] Dumenden, I.E. & English, R., 2013, ‘Fish out of water: Refugee and international’, *Journal of Inclusive Education* 17(10), 1078–1088. 10.1080/13603116.2012.732120

[CIT0022] Ellingson, L.L., 2015, ‘Embodied practices in dialysis care: On (para) professional work’, in B. Green & N. Hopwood (eds.), *The body in professional practice, learning and education*, pp. 173–189, Springer, London.

[CIT0023] Farokhi, M. & Hashemi, M., 2011, ‘The analysis of Children’s drawings: Social, emotional, physical, and psychological aspects’, *Procedia – Social and Behavioral Sciences* 30, 2219–2224. 10.1016/j.sbspro.2011.10.433

[CIT0024] Fetters, M.D. & Freshwater, D., 2015, ‘Publishing a methodological mixed methods research article’, *Journal of Mixed Methods Research* 9(3), 203–213. 10.1177/1558689815594687

[CIT0025] Garbett, D. & Tynan, B., 2010, ‘A lifeline for emerging academics’, In R.H. Cantwell & J.J. Scevak (eds.), *An academic life*, pp. 173–181, ACER Press, Camberwell.

[CIT0026] Gong, R., Wang, Q. & Shao, X., 2017, ‘Investigation on factors to influence color emotion and color preference responses’, *Optik* 36, 71–78. 10.1016/j.ijleo.2017.02.026

[CIT0027] Grant, B., 1999, ‘Walking on a rackety bridge: Mapping supervision’, in HERDSA Annual International Conference, Melbourne, 12th–15th July 1999.

[CIT0028] Grant, B., 2003, ‘Mapping the pleasures and risks of supervision’, *Discourse: Studies in the Cultural Politics of Education* 24(2), 175–190. 10.1080/0159630032000110720

[CIT0029] Grant, B., 2018, ‘Assembling ourselves differently? Contesting the dominant imaginary of doctoral supervision’, *parallax* 4(3), 356–370. 10.1080/13534645.2018.1496584

[CIT0030] Green, B., 2015, ‘Thinking bodies: Practice theory, Deleuze, and professional education’, in B. Green & N. Hopwood (eds.), *The body in professional practice, learning and education*, pp. 121–136, Springer, London.

[CIT0031] Green, B. & Hopwood, N., 2015, ‘The body in professional practice, learning and education: A question of corporeality’, in B. Green & N. Hopwood (eds.), *The body in professional practice, learning and education*, vol. 11, pp. 15–33, Springer, London.

[CIT0032] Grenfell, M., 2012, ‘Introduction’, in M. Grenfell (ed.), *Pierre Bourdieu: Key concepts*, 2nd edn., pp. 1–6, Acumen, Durnham.

[CIT0033] Guba, E. & Lincoln, Y., 1989, *Fourth generation evaluation*, Sage, Newbury Park, CA.

[CIT0034] Hall, R., 2016, *Richard Hall’s Space: Notes on desire, anxiety and academic luddism*, viewed 11 April 2021, from http://www.richard-hall.org/2016/06/01/notes-on-desire-anxiety-and-academic-luddism/.

[CIT0035] Halse, C., 2011, ‘Becoming a supervisor’: The impact of doctoral supervision on supervisors’ learning’, *Studies in Higher Education* 36(5), 557–570. 10.1080/03075079.2011.594593

[CIT0036] Halse, C. & Malfroy, J., 2009, ‘Theorising doctoral supervision as professional work’, *Studies in Higher Education* 35(1), 79–92. 10.1080/03075070902906798

[CIT0037] Hunter, J. & Hayter, M., 2019, ‘A neglected transition in nursing: The need to support the move from clinician to academic properly’, *Journal of Advanced Nursing* 75(9), 1820–1822. 10.1111/jan.14075 31115061

[CIT0038] Haplin, Y., Terry, L.M. & Curzio, J., 2017, ‘A longitudinal, mixed methods investigation of newly qualified nurses’ workplace stressors and stress experiences during transition’, *Journal of Advanced Nursing* 73(11), 2577–2586. 10.1111/jan.13344 28543602

[CIT0039] Ingham-Broomfield, R., 2017, ‘A nurses’ guide to ethical considerations and the process for ethical approval of nursing research’, *Australian Journal of Advanced Nursing* 35(1), 40–47.

[CIT0040] Irwin, S. & Winterton, M., 2011, *Debates in qualitative secondary analysis: Critical reflections*, Working Paper, Timescapes Working Paper Series, 4, University of Leeds. ISSN 1758-3349.

[CIT0041] Ivancheva, M.P., 2015, ‘The age of precarity and the new challenges to the academic profession’, *Studia Europaea* LX(1), 39–47, viewed 17 August 2021, from http://studia.ubbcluj.ro/arhiva/abstract_en.php?editie=EUROP.

[CIT0042] Ivinson, G., 2011, ‘The body and pedagogy: Beyond absent, moving bodies in pedagogic practice’, *British Journal of Sociology of Education* 33(4), 489–506. 10.1080/01425692.2012.662822

[CIT0043] Joffe, H. & Elsey, J.W., 2014, ‘Free association in psychology and the grid elaboration method’, *Review of General Psychology* 18(3), 173–185. 10.1037/gpr0000014

[CIT0044] Lawrence, L., 2020, ‘Conducting cross-cultural qualitative interviews with mainland Chinese participants during COVID: Lessons from the field’, *Qualitative Research*. Online, 0(00):1–12, 10.1177/1468794120974157

[CIT0045] Lee, A., 2018, ‘How can we develop supervisors for the modern doctorate?’, *Studies in Higher Education* 42(5), 878–890. 10.1080/03075079.2018.1438116

[CIT0046] Leibowitz, M., 1999, *A self psychological approach*, BRUNNER/MAZEL, Philadelphia, PA.

[CIT0047] Maritz, J. & Prinsloo, P., 2015, ‘A Bourdieusian perspective on becoming and being a postgraduate supervisor: The role of capital’, *Higher Education Research and Development* 34(5), 972–985. 10.1080/07294360.2015.1011085

[CIT0048] Maritz, J. & Prinsloo, P., 2019, ‘The (d)(t)oxic lifeworld of early career postgraduate supervisors’, *Teaching in Higher Education* 24(4), 563–577. 10.1080/13562517.2018.1498075

[CIT0049] McCallin, A. & Nayar, S., 2012, ‘Postgraduate research supervision: A critical review of current practice’, *Teaching in Higher Education* 17(1), 63–74. 10.1080/13562517.2011.590979

[CIT0050] McCulloch, A. & Loeser, C., 2016, ‘Does research degree supervisor training work? The impact of a professional development induction workshop on supervision practice’, *Higher Education Research & Development* 35(5), 968–982. 10.1080/07294360.2016.1139547

[CIT0051] Miles, M.B., Huberman, A.M. & Saldana, J., 2014, *Qualitative data analysis*, 3rd edn., Sage, Los Angeles, CA.

[CIT0052] Moore, R., 2013, ‘Capital’, in M. Grenfell (ed.), *Piere Bourdieu key concepts*, pp. 101–117, Acumen, Durham.

[CIT0053] Mulcohy, D., 2015, ‘Body matters: The critical contribution of affect in school classrooms and beyond’, in B. Green & N. Hopwood (eds.), *The body in professional practice, learning and education*, pp. 105–120, Springer, London.

[CIT0054] Naidoo, J.R. & Mthembu, S., 2015, ‘An exploration of the experiences and practices of nurse academics regarding postgraduate research supervision at a South African university’, *African Journal of Health Professions Education* 7(2), 216–219. 10.7196/AJHPE.443

[CIT0055] Philpott, C., 2015, ‘Creating an in-school pastoral system for student teachers in school-based initial teacher education. Pastoral Care in Education’, *An International Journal of Personal, Social and Emotional Development* 33(1), 8–19. 10.1080/02643944.2014.990989

[CIT0056] Reay, D., 2004, It’s all becoming a habitus’: Beyond the habitual use of habitus in educational research’, *British Journal of Sociology of Education* 25(4), 433–444. 10.1080/0142569042000236934

[CIT0057] Reay, D., 2015, ‘Habitus and the psychosocial: Bourdieu with feelings’, *Cambridge Journal of Education* 45(1), 9–23. 10.1080/0305764X.2014.990420

[CIT0058] Rhem, J., 2013, *Before and after students ‘get it’: Threshold concepts*, viewed 09 April 2021, from https://teachingcommons.stanford.edu/teaching-talk/and-after-students-get-it-threshold-concepts.

[CIT0059] Richards, L., 2015, *Handling qualitative data*, 3rd edn., Sage, Los Angeles, CA.

[CIT0060] Richardson, L. & St. Pierre, E.A., 2005, ‘Writing: A method of inquiry’, in N.K. Denzin & Y.S. Lincoln (eds.), *The Sage handbook of qualitative research*, pp. 959–978, Sage, Thousand Oaks, CA.

[CIT0061] Reilly, R.C., 2013, ‘Found poems, member checking, and crises of representation’, *Qualitative Report* 18(15), 1–18.

[CIT0062] Riseborough, G., 1981, ‘Teacher careers and comprehensive schooling: An empirical study’, *Sociology* 15(3), 352–380. 10.1177/003803858101500303

[CIT0063] Robinson, R.C., 2014, ‘Sampling in interview-based qualitative research: A theoretical and practical guide’, *Qualitative Research in Psychology* 11(1), 25–41. 10.1080/14780887.2013.801543

[CIT0064] Smart, F., 2014, ‘Poetic transcription: An option in supporting the early career academic?’, *Journal of Perspectives in Applied Academic Practice* 2(3), 66–70. 10.14297/jpaap.v2i3.114

[CIT0065] Stacy, S.J. & Hinrichs, M., 2017, ‘Big tent criteria for qualitative research’, The International Encyclopedia of Communication Research Methods, in Jörg Matthes (General Editor), *The international encyclopedia of communication research*, pp. 134–143, Wiley-Blackwell, Hoboken, NJ. 10.1002/9781118901731.iecrm0016

[CIT0066] Sutton, T.M. & Altarriba, J., 2016, ‘Finding the positive in all of the negative: Facilitation for color-related emotion words in a negative priming paradigm’, *Acta Psychologica* 170, 84–93. 10.1016/j.actpsy.2016.06.012 27380622

[CIT0067] Tachuchi, H.L., 2011, ‘Investigating learning, participation and becoming in early childhood practices with a relational materialist approach’, *Global Studies of Childhood* 1(1), 36–50. 10.2304/gsch.2011.1.1.36.

[CIT0068] Trowler, P.R., 1998, *Academics responding to change. New higher education frameworks and academic cultures*, Open University, Philadelphia, PA.

[CIT0069] Wacquant, L., 2016, ‘A concise genealogy and anatomy of habitus’, *The Sociological Review* 64(1), 64–72. 10.1111/1467-954X.12356

[CIT0070] Webbstock, D. & Sehoole, C., 2016, ‘Academic staffing’, Council on Higher Education (CHE), in *South African Higher education Review: Two decades of democracy*, pp. 279–315, Council on Higher Education, Pretoria.

[CIT0071] World Health Organization (WHO), 2021, *Mental health and Covid-19*, viewed 09 April 2021, from https://www.who.int/teams/mental-health-and-substance-use/covid-19.

